# Variance-resistant PTB7 and axially-substituted silicon phthalocyanines as active materials for high-Voc organic photovoltaics

**DOI:** 10.1038/s41598-021-94704-5

**Published:** 2021-07-28

**Authors:** Mario C. Vebber, Nicole A. Rice, Jaclyn L. Brusso, Benoît H. Lessard

**Affiliations:** 1 Department of Chemical and Biological Engineering, University of Ottawa, 161 Louis Pasteur, Ottawa, ON K1N 6N5 Canada; 2 Department of Chemistry and Biomolecular Sciences, University of Ottawa, 150 Louis Pasteur, Ottawa, ON K1N 6N5 Canada; 3 School of Electrical Engineering and Computer Science, University of Ottawa, 800 King Edward, Ottawa, ON K1N 6N5 Canada

**Keywords:** Materials science, Materials for devices, Materials for energy and catalysis, Engineering, Chemical engineering, Electrical and electronic engineering, Energy infrastructure

## Abstract

While the efficiency of organic photovoltaics (OPVs) has improved drastically in the past decade, such devices rely on exorbitantly expensive materials that are unfeasible for commercial applications. Moreover, examples of high voltage single-junction devices, which are necessary for several applications, particularly low-power electronics and rechargeable batteries, are lacking in literature. Alternatively, silicon phthalocyanines (R_2_-SiPc) are inexpensive, industrially scalable organic semiconductors, having a minimal synthetic complexity (SC) index, and are capable of producing high voltages when used as acceptors in OPVs. In the present work, we have developed high voltage OPVs composed of poly({4,8-bis[(2-ethylhexyl)oxy]benzo[1,2-b:4,5-b′]dithiophene-2,6-diyl}{3-fluoro-2-[(2-ethylhexyl)carbonyl] thieno [3,4 b]thiophenediyl}) (PTB7) and an SiPc derivative ((3BS)_2_-SiPc). While changes to the solvent system had a strong effect on performance, interestingly, the PTB7:(3BS)_2_-SiPc active layer were robust to spin speed, annealing and components ratio. This invariance is a desirable characteristic for industrial production. All PTB7:(3BS)_2_-SiPc devices produced high open circuit voltages between 1.0 and 1.07 V, while maintaining 80% of the overall efficiency, when compared to their fullerene-based counterpart.

## Introduction

Organic photovoltaics (OPVs) are a promising solar energy technology with the potential for low manufacturing cost and quick energy payback^1,2^. OPVs can be fabricated by solution-based techniques, such as spin-coating, blade-coating and a variety of printing techniques, facilitating their integration into continuous, high throughput processing, which is unachievable with traditional silicon technology^1,3–5^. In the past 5 years, single-junction OPVs with record power conversion efficiencies (PCE) of 15–19% have been reported^6–9^, approaching commercial solar cell performances. However, these high efficiencies are achieved using expensive small molecules and polymers, the production of which is not scalable as they require complex multi-step synthesis and purification methods^10–14^. Most of these state-of-the-art OPVs often excel in high current densities (*Jsc*) and fill factor (FF), while providing middling open-circuit voltage (*Voc*), between 0.7 and 0.8 V^6–9,15^.

High-voltage OPVs are of great interest for certain applications, particularly in rechargeable batteries and low-power electronics, which always require a minimum voltage to operate^16–18^. While high *Voc*s can be obtained by tandem cells or cells in series, this requires all cells to produce similar currents, which is virtually impossible to achieve in applications where lighting is inhomogeneous^16^. Some ternary BHJs with *Voc*s above 0.9 V have been reported^19,20^, albeit relying on high-cost, non-scalable materials. It remains that the *Voc* of the majority of single-junction photovoltaics rarely surpasses 0.8 V, including silicon-based devices. Recently, a few research groups have used simple and low-cost small molecules as non-fullerene acceptors (NFAs) in OPVs, and achieved *Voc* ≥ 1.0 V^12,21–24^. Such architectures may hold the key for commercial viability as they can be manufactured on an industrial scale, provided that the efficiency and stability of the devices is sufficiently high^21,25^.

Axially substituted silicon phthalocyanines (R_2_-SiPc) are ideal candidates for low-cost, high- *Voc* acceptor materials^24,26,27^. While metal phthalocyanine (MPc) have been investigated in organic electronic applications for more than 50 year, R_2_-SiPcs are relatively understudied, having emerged in recent years^28^ and successfully incorporated in multiple new application, including organic thin-film transistors (OTFTs)^29–34^, organic light-emitting diodes (OLEDs)^26,35,36^ and in OPVs^24,26,37–40^. The synthetic complexity (SC) index^41^ of R_2_-SiPcs have been calculated to be at least three times lower (SC = 12)^27^ than that of several prominent OPV acceptors materials, such as PC_61_BM (SC = 36)^42^, Y6 (SC = 59)^43^ and ITIC (SC = 67)^44^. The exceptionally low SC index of R_2_-SiPcs makes them exceedingly promising organic semiconductors for OPVs. Historically, R_2_-SiPcs have mainly been employed as ternary additives in OPVs^37–40,45^. However, in a recent study by Grant et al., an OPV composed of a blend of R_2_-SiPc and poly-3-hexylthiophene (P3HT) achieved higher *Voc* and PCE than the fullerene-based analogue. Remarkably, when paired with poly[[4,8-bis[5-(2-ethylhexyl)-2-thienyl]benzo[1,2-b:4,5-b′]dithiophene-2,6-diyl]-2,5-thiophenediyl [5,7-bis(2-ethylhexyl)-4,8-dioxo-4H,8H-benzo[1,2-c:4,5-c′]dithiophene-1,3-diyl]] (PBDB-T), yielded a device with an exceptionally high *Voc* of nearly 1.1 V^24^. Additionally, relatively simple chemical modification of R_2_-SiPcs can address other common issues in OPVs, such as stability, by including cross-linking groups in the SiPc structure to improve the stability of the active layer’s nanostructure^46^. Nonetheless, there are relatively few reports investigating the use of R_2_-SiPc as stand-alone acceptors in OPVs^24,26,27^. therefore it is vital to investigate pairing these cost-effective molecules with different donor polymers and optimizing these devices to exploit their full potential in OPVs.

**Figure 1:**
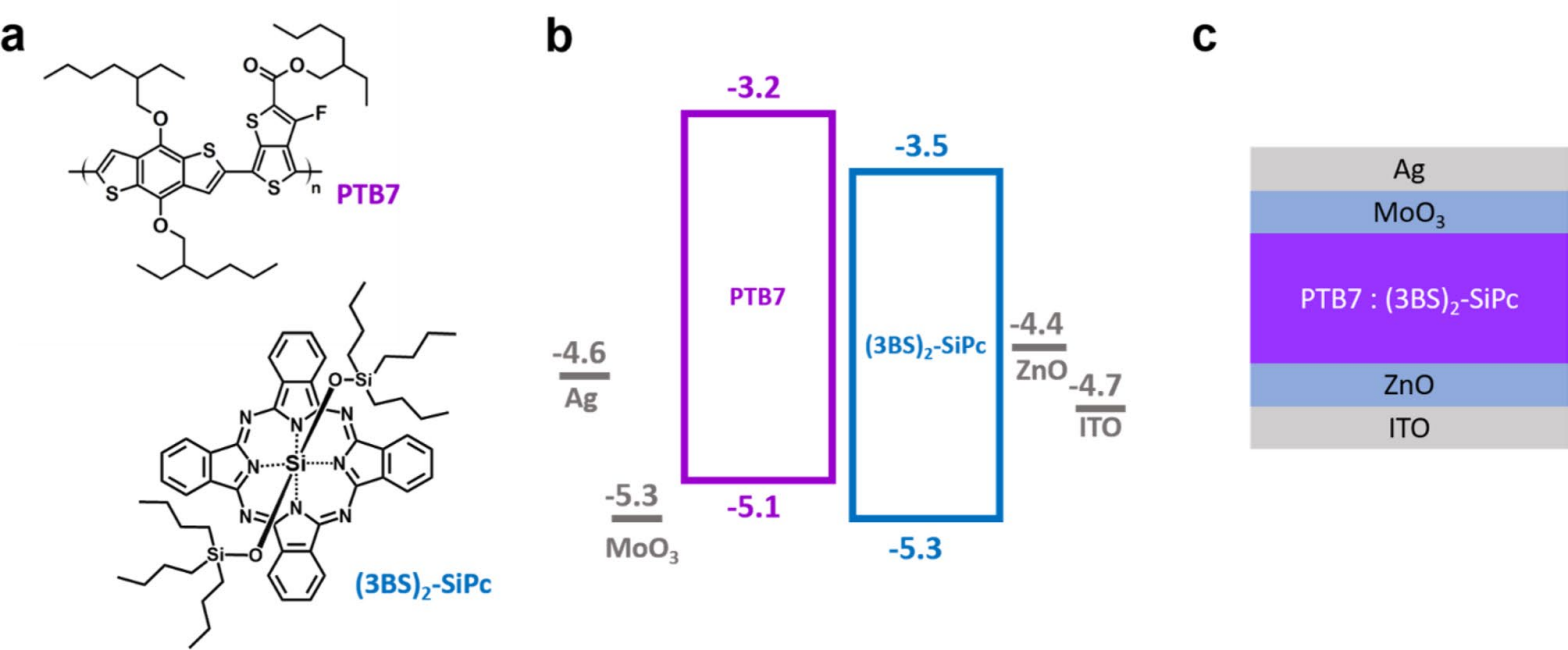
(**a**) Chemical structure of donor and acceptor materials; (**b**) energy level diagram of the device components; and (**c**) schematic representation of device architecture.

In the present work we have fabricated OPVs using poly[[4,8-bis[(2-ethylhexyl)oxy]benzo[1,2-b:4,5-b']dithiophene-2,6-diyl][3-fluoro-2-[(2-ethylhexyl) carbonyl] thieno[3,4-b]thiophenediyl]] (PTB7, Fig. 1) and bis(tri-n-butylsilyl oxide) silicon phthalocyanine ((3BS)_2_-SiPc; Fig. 1). Our group has recently reported similar devices, by pairing ((3BS)_2_-SiPc with PBDB-T, and here we continue to explore this NFA with PTB7, another high performing polymer, that possesses more adequate energy level alignment with respect to (3BS)_2_-SiPc (Fig. 1).These devices were characterized by atomic force microscopy (AFM) and external quantum efficiency (EQE). The OPVs were optimized and yielded devices with a high *Voc* of 1.05 V, while maintaining 80% of the overall PCE, when compared to a fullerene-based analogue.

## Experimental section

### Materials

Two different molecular weights of PTB7 (93 kg mol^−1^, PDI 2.6 and 16 kg mol^−1^, PDI 2.4) was purchased from 1-Material and used as received. Bis(tri-n-butyl siloxy) silicon phthalocyanine ((3BS)_2_-SiPc) was synthesized and purified according to the literature^45^. Dichlorobenzene (DCB, 99%), chlorobenzene (CB, 99%), chloroform (CF, 99%), dichloromethane (DCM, 99%), diiodooctane (DIO, 98%) and diphenylether (DPE, 99%), zinc acetate dehydrate (Zn(Ac)_2_ ∙ 2 H_2_O, 99%), ethylamine (97%) were all purchased from Sigma-Aldrich and used without further purification. Ag (99.99%) was purchased from Angstrom Engineering Inc. and MoO_3_ (99.99%) was purchased from Strem.

### Devices

Indium-tin-oxide (ITO) coated glass substrates (100 nm, 20 Ω/sq, 1 in by 1 in), purchased from Thin Film Devices Inc., were cleaned in an ultrasound bath sequentially with soapy water, DI water, acetone (99%) and methanol (95%) to remove any debris. The ITO slides were then dried with a N_2_ jet and placed in an air plasma cleaner for 15 min to remove any residual organics. The zinc oxide (ZnO) electron-transport layer was deposited by spin coating 150 µl of an ethanolic solution of Zn(Ac)_2_ ∙ 2 H_2_O (3.3%) and ethanolamine (0.9%) at 2000 RPM, followed by annealing for 1 h in air at 180 °C. The substrates were then move into a N_2_ glovebox where the remainder of the procedure was carried out. Preparation of the active layer was achieved using a variety of conditions, details of which are provided in Table 1. The active layer components are illustrated in Fig. 1a. After deposition, the films were dried in the glovebox, at room temperature, for 1 h before being transferred to an evaporation chamber (Angstrom EvoVac), where MoO_3_ (7 nm) and silver electrodes (70 nm) were deposited by physical vapor deposition at a pressure below 10^−6^ torr, to yield 5 individual 0.32 cm^2^ devices per substrate, as defined by shadow masks. Energy level diagram and device architecture are shown in Fig. 1b and c, respectively. Device performance was characterized using a custom push pin probe station connected to multiplexer and Keithley 2400 source meter. All OPVs were assessed under 1000 W m^−2^ light intensity, provided by a solar simulator (Xenon lamp, AM1.5) and scanned between − 2.0 and 2.0 V. Light intensity was verified using an NREL certified silicon standard cell prior to every run. Series and shunt resistances have been calculated from the slope of the IV curves, at the *Jsc* and *Voc*, respectively. External quantum efficiency (EQE) plots were recorded using a Newport Quantx-300 instrument outside of the glovebox. Prior to EQE measurements, the devices were encapsulated in an epoxy resin (Norland NOA61) cured under UV-light. Atomic force microscopy (AFM) topography images of the active layer films were obtained using a Bruker Dimension Icon instrument, with ScanAsyst-Air probes in tapping mode, at a frequency of 0.8 Hz; image processing was performed with NanoScope Analysis v1.8.

**Table 1:** Experimental conditions and resulting performance of PTB7:(3BS)_2_-SiPc BHJ OPV devices.

#	D:A Ratio	Solvent	Spin speed: Annealing (RPM: °C/min)	Voc^a^^)^ (V)	Jsc^a^^)^ (mA cm^−2^)	FF^a^^)^	PCE^a^^)^ (%)
1	1:1.5^b)^	CB	1500: N/A	0.76 ± 0.01	12.57 ± 0.3	0.49 ± 0.02	4.73 ± 0.03
2	1:1.5^b)^	CB + DIO (3%) + DPE (2%)	1500: N/A	0.70 ± 0.01	14.66 ± 0.09	0.65 ± 0.01	6.67 ± 0.09
3	1:1.5^c)^	CB	2000: N/A	1.05 ± 0.01	7.66 ± 0.08	0.45 ± 0.01	3.64 ± 0.05
4	1:1.5^c)^	CB	1500: N/A	1.06 ± 0.004	7.61 ± 0.04	0.45 ± 0.01	3.59 ± 0.01
5	1:1.5^c)^	CB	1000: N/A	1.06 ± 0.004	7.51 ± 0.05	0.43 ± 0.01	3.49 ± 0.01
6	1:1.5	CB	1500: 100/15	1.07 ± 0.01	7.58 ± 0.04	0.46 ± 0.01	3.55 ± 0.03
7	1:1.5	CB	1500: 100/30	1.00 ± 0.1	7.31 ± 0.05	0.47 ± 0.02	3.41 ± 0.2
8	1:1.5	CB	1500: 150/15	1.06 ± 0.01	7.11 ± 0.05	0.46 ± 0.02	3.48 ± 0.2
9	1:1.5	CB	1500: 150/30	1.03 ± 0.01	6.88 ± 0.09	0.44 ± 0.01	3.11 ± 0.08
10	1:1	CB	1500: N/A	1.06 ± 0.01	7.24 ± 0.04	0.44 ± 0.01	3.38 ± 0.02
11	1:1.5	CB	1500: N/A	1.05 ± 0.01	7.68 ± 0.2	0.46 ± 0.01	3.76 ± 0.1
12	1:1.8	CB	1500: N/A	1.05 ± 0.01	7.68 ± 0.07	0.48 ± 0.01	3.82 ± 0.04
13	1:2	CB	1500: N/A	1.04 ± 0.01	7.59 ± 0.03	0.48 ± 0.01	3.79 ± 0.03
14	1:1	DCB	1500: N/A	1.02 ± 0.004	7.48 ± 0.09	0.40 ± 0.01	3.06 ± 0.01
15	1:1.8	CB:CF 3:1	1500: N/A	1.04 ± 0.01	7.84 ± 0.09	0.45 ± 0.01	3.68 ± 0.04
16	1:1.8	CB:DCM 3:1	1500: N/A	1.06 ± 0.01	7.81 ± 0.05	0.45 ± 0.01	3.73 ± 0.01
17	1:1.8	CF	3000: N/A	1.02 ± 0.01	4.67 ± 0.1	0.38 ± 0.01	1.79 ± 0.08
18	1:1.5	CB + DIO (3%) + DPE (2%)	1500: N/A	No functioning device			
19	1:1.8^d)^	CB	1000: N/A	1.04 ± 0.004	5.84 ± 0.05	0.41 ± 0.01	2.47 ± 0.02
20	1:1.8^d)^	CB	1500: N/A	1.04 ± 0.004	6.19 ± 0.07	0.43 ± 0.01	2.74 ± 0.05

^a^^)^All BHJ OPVs were assessed under 1000 W m^−2^ light intensity, and scanned between − 2.0 and 2.0 V. The open circuit voltage (*Voc*), short circuit current (*Jsc*), fill factor (FF) and the power conversion efficiency (PCE) were obtained from an average of 4–8 devices with an individual area 0.32 cm^2^ per device. Devices that have not been annealed are marked as “N/A”.

^b)^Baseline device composed of PTB7:PC_61_BM at a 1:1.5 ratio.

^c)^Solution was not filtered before deposition.

^d)^PTB7 with M_w_ of 96 kg mol^−1^ was used for all devices except device 19 and 20 where a M_w_ of 16 kg mol^−1^ was used.

## Results and discussion

Bulk heterojunction (BHJ) OPV devices (Fig. 1c) were fabricated by combining PTB7 (donor) with (3BS)_2_-SiPc (acceptor, Fig. 1a). The energy levels of PTB7, (3BS)_2_-SiPc and the other materials in the BHJ OPV devices are shown in Fig. 1b. Extensive optimization of the active layer was performed, as shown in Table 1. The optimized parameters were spin-rate, annealing, donor:acceptor ratio and choice of solvent. Parameters including spin-rate, annealing time and temperature, donor:acceptor ratio, and choice of solvent were all investigated.

The best PTB7:(3BS)_2_-SiPc BHJ OPV device (**12**) was obtained with an excess of (3BS)_2_-SiPc (1:1.8 ratio) in CB, at a spin rate of 1500 RPM, resulting in a high *Voc* of 1.05 ± 0.01 V, a modest *J*_*SC*_ of 7.68 ± 0.07 mA cm^−2^, fill factor (*FF*) of 0.48 ± 0.01 and an overall PCE of 3.82 ± 0.04 (Table 1). While benchmark OPVs often have *FF* between 0.6 and 0.7^47^, these values are comparable to those of the PTB7:PC_61_BM baseline device prepared (**1**). The average performance of device **12** is comparable to the champion device in the series, which achieved a PCE of 3.85% (*Voc* = 1.06 V*, J*_*SC*_ = 7.71 mA·cm^−2^, FF = 0.47), within one standard deviation. These results are also slightly superior to the high-*Voc* devices previously reported by our group, based on PBDB-T/:(3BS)_2_-SiPc devices (PCE = 3.4%)^24^. This improvement comes from a 10% improvement in current density, which can be attributed to the favourable energy level alignment between PTB7 and (3BS)_2_-SiPc, with a 0.2–0.3 eV separation between HOMO and LUMO levels (Fig. 1). This energy gap facilitates charge separation and can lead to greater currents.

**Figure 2:**
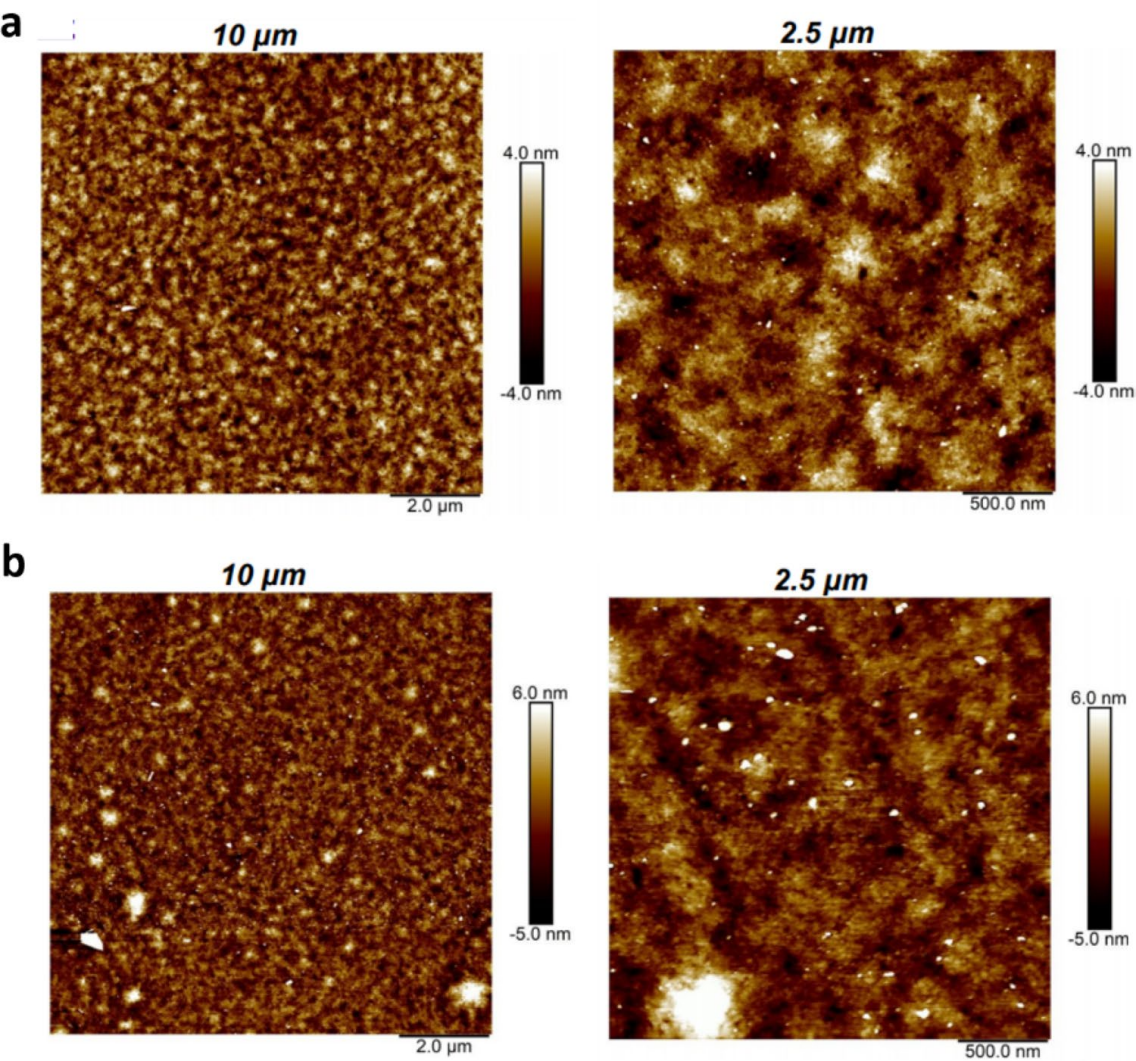
AFM height images of the PTB7:(3BS)_2_-SiPc films on two different scales (10 and 2.5 µm (**a**) before annealing and (**b**) after annealing (15 min at 100 °C).

When optimizing the PTB7:(3BS)_2_-SiPc BHJ OPV devices we found that PCE improves only slightly when deposition spin rate is increased from 1000 to 2000 RPM (devices **3**–**5**), demonstrating that the PTB7:(3BS)_2_-SiPc active layer can be successfully deposited at different spin-rates. Interestingly, film thickness remains relatively constant, decreasing slightly from 129 to 115 nm, when varying spin rate from 1000 to 2000 RPM, which does not follow the typical spin-coating equation, that predicts a thickness variation of nearly 50% between the two films^48^. This behaviour suggests a strong dilatant characteristic of the PTB7:(3BS)_2_-SiPc solution, resisting the centrifugal force. While dilatant behaviour is common in polymer solutions, the extent of the effect observed here suggest strong interaction of PTB7 with the (3BS)_2_-SiPc moieties in solution, leading to rheological modification. In general, annealing also showed a weak effect on device PCE (devices **6**–**9**), slightly increasing FF while decreasing *Jsc*. Figure 2 shows AFM images before (Fig. 2a) and after annealing for 15 min at 100 °C (Fig. 2b). Comparison of the films at various length scales shows that some larger amorphous features are formed during the annealing step, which is reflected in the increased roughness of the films: *r*_*q*_ = 1.31 nm before annealing and *r*_*q*_ = 2.0 nm after. Nonetheless, in general overall surface morphology and height features are mostly retained. Longer annealing times were slightly detrimental to device performance, most likely due to disruption of the active layer through formation of large agglomerates, as suggested in Fig. 2b. The (3BS)_2_-SiPc to PTB7 ratio (devices **10**–**13**) only had a noticeable effect on the PCE when the ratio was reduced below 1.5, which is observed when comparing devices **10** and **11**, with ratios of 1.0 and 1.5, respectively. Alternatively, devices **11**, **12** and **13,** with ratios of 1.5, 1.8 and 2.0, respectively, have remarkably similar performances. While PTB7:PC_61_BM devices have been optimized at a 1:1.5 ratio^49^, the PTB7:(3BS)_2_-SiPc BHJ OPV devices had the best performance when using a 1:1.8 ratio (Table 1). In summary, we found that the (3BS)_2_-SiPc:PTB7 blend is fairly invariant to spin rate, acceptor:donor ratio and annealing conditions. This low variability is a desirable property for high throughput manufacture, where minor variations are inevitable.

Alternatively, modification of the active layer solvent system had a significant impact on device performance. Solvent additives, namely DIO and DPE, have been previously reported to play a critical role in the film morphology of the active layer in PTB7:PC_61_BM devices, resulting in significant improvements in current density and PCE^50^. Such high boiling point additives promote higher crystallinity and improved nanomorphology in PTB7:fullerene blends^50^, typically resulting in increases of more than 40% in PCE. We have observed these improvements as well in our baseline PTB7:PC_61_BM baselines (**1** and **2,** Table 1). However, when incorporating these additives in the fabrication of PTB7:(3BS)_2_-SiPc BHJ OPV devices (device **18**), we were unable to obtain functioning devices. This may be attributed to (3BS)_2_-SiPc, which has been reported to crystallize rapidly^46,51,52^, and the use of high boiling point additives thereby exacerbating this behaviour, creating micrometric or even submillimetric domains (patterns are visible to the naked eye) as opposed to the nanometric phase separation required for functioning OPVs^53,54^. In attempt to counter this crystallization we explored the use of low-boiling point solvent additives, such as DCM and CF (devices **15** and **16**) but these solvent changes led to negligible improvements in performance. Using CF as a single low-boiling point solvent (device **17**) resulted in thicker films due to rapid evaporation, which approximately halved the PCE when compared to the best devices deposited with solutions in CB. We have also investigated if incorporating a low a low-M_w_ PTB7 (16 kg mol^−1^) instead of the conventional PTB7 (M_w_ = 96 kg mol^−1^) could improve the crystallinity of the P7B7 phase without additives. Functioning devices were obtained (**19** and **20**), but with lower efficiency compared to the optimized device achieved from low-M_w_ PTB7 (**12**).

While the *Jsc* and FF of the PTB7:(3BS)_2_-SiPc BHJ OPV devices were modest, it is important to note the consistently high *Voc* between 1.00 and 1.07 V, which is a very desirable and often rare characteristic in OPVs. Note that current state-of-the-art OPVs typically display *Voc* values around 0.8V^7–9^. The high voltages can be attributed to the large difference between the energy levels of the donor’s highest occupied molecular orbital (HOMO) and the acceptor’s lowest unoccupied molecular orbital (LUMO), as illustrated in Fig. 1b.

Table 2 compares the series and shunt resistances of our optimized device **12** with literature-based devices containing either PTB7 or P3HT as the donor and PC_61_BM or (3BS)_2_-SiPc as the acceptor. Strikingly, the shunt resistance of the PTB7:(3BS)_2_-SiPc BHJ OPV device is significantly lower than the others, which indicates a high rate of charge recombination in the active layer film^1,47^ and may offer a potential explanation for the relatively low *Jsc* compared to PTB7:PC_61_BM devices (Table 1, 2). This may be ascribed to the small energy level offset only 0.2–0.3 eV between the donor and acceptor, which could be impairing the dissociation of excitons at the PTB7:(3BS)_2_-SiPc interface^1^. Moreover, AFM images (Fig. 2) show that domain sizes are in the hundreds of nm range, which is often too large for optimal BHJ OPV operation, given the average distance travelled by excitons before recombination is around 5–15 nm^1,54,55^.

**Table 2:** Thickness, series and shunt resistances of the optimized devices and relevant comparative devices.

Device	Series (Ω)	Shunt (Ω)	Thickness (nm)
P3HT:PC_61_BM^45^^a)^	11	1676	200
P3HT:(3BS)_2_-SiPc^24^^a)^	15	816	100
PTB7:PC_61_BM (**1**)	9	1352	90
PTB7:(3BS)_2_-SiPc (**12**)	22	463	115

^a)^Devices have been previously reported and further characterized here.

**Figure 3:**
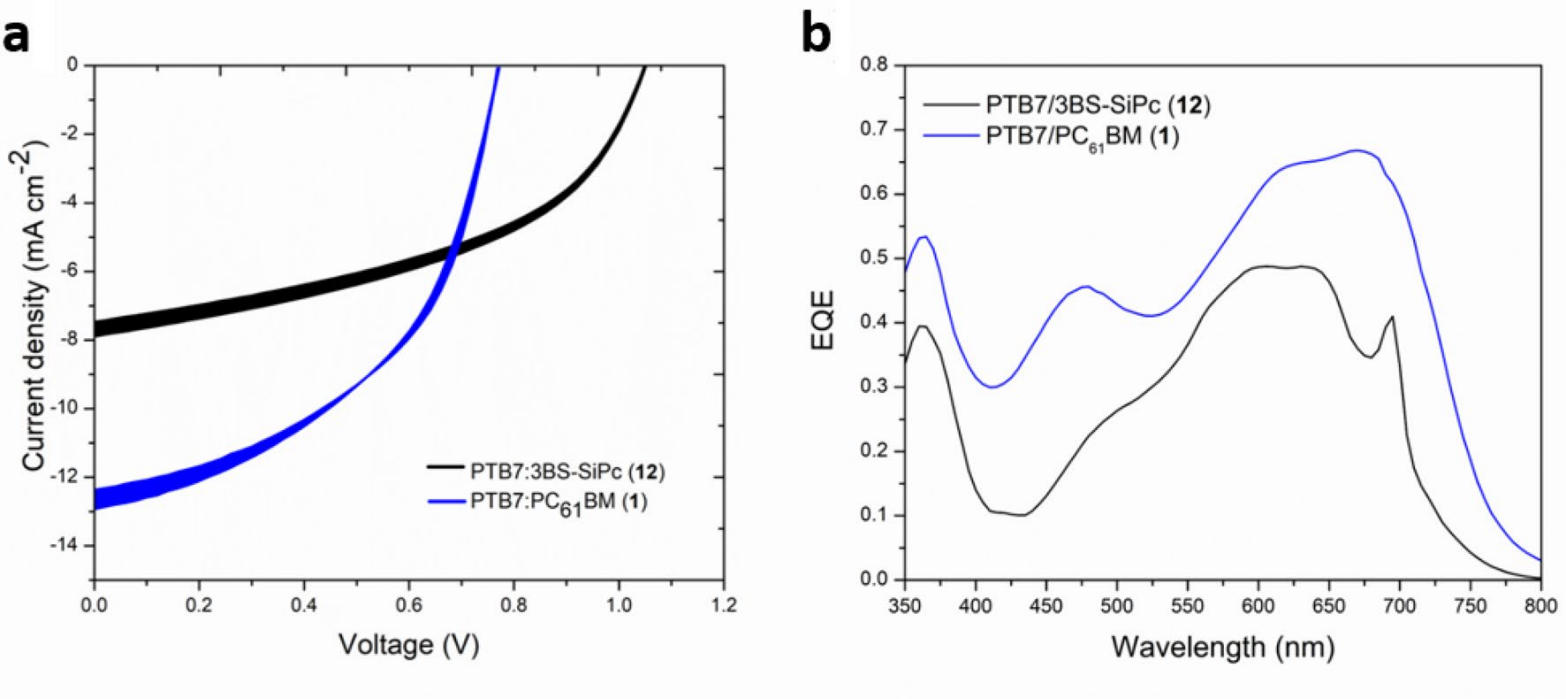
OPV characteristics (**a**) I–V curves, where line thickness corresponds to the standard deviation of 4 devices, and (**b**) EQE spectra of the optimized device (**12**) and the fullerene-containing baseline (**1**).

Figure 3a shows the I–V curves for the optimized PTB7:(3BS)_2_-SiPc device (**12**) compared to the PTB7:PC_61_BM baseline (**1**) and Fig. 3b displays the EQE curves for the same devices. While (3BS)_2_-SiPc provides some extra light absorption around 700 nm, the overall quantum efficiency is lower than that of the fullerene-based device. This trade off is often seen in SiPc-based devices^24^. Both devices had similar shelf-life, retaining approximately 92% of their initial PCE after being stored for 6 months in N_2_. Moreover, the EQE spectrum of device **12** is slightly blue-shifted and sharper in comparison to the baseline (**1**), which is associated with smaller crystallinity of the polymeric phase^50,56^. This suggests that (3BS)_2_-SiPc inhibits the crystallization of PTB7, and it is one of the causes for the lower current observed when compared to the fullerene baseline. The lower crystallinity of the polymeric network can also impair charge conduction and contribute to the low shunt resistance measured for such devices. We surmise that a new type of additive, that simultaneously promotes the crystallization of PTB7 while keeping the SiPc domain size small, could push the efficiency of this class of devices beyond that of the PTB7:PC_61_BM baseline, but the authors are not familiar with any additive that fits these requirements.

R_2_-SiPcs continue to show promise as NFAs in BHJ OPVs. However, their tendency to quickly crystallize and their shallow LUMO level have been detrimental to achieving high performance. Moving forward, the chemical versatility of R_2_-SiPc molecules, will facilitate fine tuning of the material properties such as their frontier orbital energy levels and the solid-state packing properties, providing the potential for improving device performance while still remaining synthetically simple molecules to produce.

## Conclusion

We paired PTB7, a high performing donor polymer, with a low cost and easy to synthesize acceptor (3BS)_2_-SiPc in OPVs and observed a significant improvement in *Voc* values. Device performance was robust to changes in the spin speed, acceptor:donor ratio and annealing; although this hinders a route towards device optimization, it is ultimately a desirable property for high throughput fabrication of OPVs. When replacing the fullerene acceptor with (3BS)_2_-SiPc, 80% of the overall device efficiency was retained, while a high average *Voc* of 1.05 V was otained. These findings further establish SiPc-based acceptors as promising NFA candidates for high voltage OPV devices. Control of the crystallization of the SiPc will be key to yield the desired nanomorphology in the active layer will be key in the development of high performing device.
